# A map of bat virus receptors derived from single-cell multiomics

**DOI:** 10.1038/s41597-022-01447-7

**Published:** 2022-06-14

**Authors:** Tianhang Lv, Xiaoshan Wang, Chao Yu, Zhifeng Wang, Rong Xiang, Linmiao Li, Yue Yuan, Yuhang Wang, Xiaoyu Wei, Yeya Yu, Xiangyang He, Libiao Zhang, Qiuting Deng, Peiying Wu, Yong Hou, Jinping Chen, Chuanyu Liu, Gary Wong, Longqi Liu

**Affiliations:** 1 College of Life Sciences, University of Chinese Academy of Sciences, Beijing, 100049 China; 2 BGI-Shenzhen, Shenzhen, 518083 China; 3 CAS Key Laboratory of Molecular Virology and Immunology, Institut Pasteur of Shanghai, Chinese Academy of Sciences, Shanghai, 200031 China; 4 Shenzhen Key Laboratory of Single-Cell Omics, BGI-Shenzhen, Shenzhen, 518120 China; 5 Guangdong Key Laboratory of Animal Conservation and Resource Utilization, Guangdong Public Laboratory of Wild Animal Conservation and Utilization, Institute of Zoology, Guangdong Academy of Sciences, Guangzhou, 510260 China; 6 School of Biology and Biological Engineering, South China University of Technology, Guangzhou, 510006 China; 7 BGI College, Zhengzhou University, Zhengzhou, 450000 China; 8 Shenzhen Bay Laboratory, Shenzhen, 518083 China

**Keywords:** Viral infection, Infection

## Abstract

Bats are considered reservoirs of many lethal zoonotic viruses and have been implicated in several outbreaks of emerging infectious diseases, such as SARS-CoV, MERS-CoV, and SARS-CoV-2. It is necessary to systematically derive the expression patterns of bat virus receptors and their regulatory features for future research into bat-borne viruses and the prediction and prevention of pandemics. Here, we performed single-nucleus RNA sequencing (snRNA-seq) and single-nucleus assay for transposase-accessible chromatin using sequencing (snATAC-seq) of major organ samples collected from Chinese horseshoe bats (*Rhinolophus affinis*) and systematically checked the expression pattern of bat-related virus receptors and chromatin accessibility across organs and cell types, providing a valuable dataset for studying the nature of infection among bat-borne viruses.

## Abstract (summary)

**Table:** 

Measurement(s)	RNA-seq gene expression profiling assay • ATAC-Seq
Technology Type(s)	RNA-seq of coding RNA from single cells • Single cell ATAC-seq (cell index)
Sample Characteristic - Organism	Rhinolophus affinis

## Background & Summary

Bats are one of the most diverse mammalian groups, comprising approximately one-fifth of all known mammal species. Bats have been identified as natural reservoir hosts of several emerging viruses that can cause severe disease in humans, including Ebola virus disease and Nipah fever^1,2^. Accumulating evidence also suggests that other emerging viruses, such as severe acute respiratory syndrome coronavirus (SARS-CoV) and Middle East respiratory coronavirus (MERS-CoV), also have bat origins^3^. Another emerging coronavirus, swine acute diarrhea syndrome coronavirus, emerged from horseshoe bats and killed many pigs^4^. The COVID-19 pandemic caused by severe acute respiratory syndrome coronavirus 2 (SARS-CoV-2) further underscores the ongoing threat of bat-borne virus spillover^5^. The shedding of these zoonotic viruses from bat populations can vary considerably across locations and times, posing fluctuating threats of spillover to other species. Transmission is promoted by successive processes that enable an animal pathogen to establish an infection in a human. The probability of zoonotic spillover is determined by interactions among several factors, including disease dynamics in the reservoir host, pathogen exposure, and genetic factors that affect host susceptibility to infections. One of the most essential and key characteristics of zoonotic spillover is virus-receptor interaction^6^. As viruses only replicate inside living cells, these pathogens have to cope with a series of positive and negative factors in the target cells to survive. In the absence of an appropriate receptor on the cells, they cannot achieve infection and therefore cannot replicate. Moreover, the presence or absence of specific cell surface receptors can influence the host range and tissue tropism of viruses^7^. Understanding virus receptor patterns *in vivo* would be an important first step for preventing and responding to future outbreaks.

Single-nucleus RNA sequencing (snRNA-seq) is now widely used in different species, such as humans^8,9^ and mice^10^, to deconstruct the composition of organs and detect cell-type subgroups in tissues. snRNA-seq can also be used to profile gene expression patterns across tissues, organs, and even the whole body^11^. Thus, it is feasible to characterize the *in vivo* expression pattern of bat-related virus receptors using snRNA-seq. Single-nucleus assay for transposase-accessible chromatin using sequencing (snATAC-seq) has recently been developed to study cell-type-specific chromatin accessibility in tissue or organ samples containing a heterogeneous cellular population^12^, and such data can be used to explore gene regulatory networks^13^. Here, we performed snATAC-seq to study the chromatin accessibility and regulatory features of bat virus receptors.

In this study, we performed snRNA-seq and snATAC-seq in the intermediate horseshoe bat (*Rhinolophus affinis*), a species of the *Rhinolophidae* family that is widely used in bat virus research^3,14,15^. We performed snRNA-seq of seven organs, including the brain, heart, kidney, lung, spleen, liver, and stomach, in a total of 85,832 nuclei. We also performed snATAC-seq of two organs, the kidney and lung, in a total of 12,678 nuclei. We profiled the expression pattern of bat-related virus receptors across these seven organs based on DBatVir, a database of bat-associated viruses that lists over 4,100 bat-associated animal viruses from 23 virus families^16^, showing that many of these receptors present organ- and cell-type-specific expression. Meanwhile, we checked the chromatin accessibility of virus receptors in the kidney and lung and found that their expression patterns were different among organs and tissues.

## Methods

### Sample collection

*Rhinolophus affinis* was obtained from a cave in Guangdong Province during April 2020. The bats were treated with pentobarbital sodium (75 mg/kg). The brain, heart, lung, kidney, stomach, liver, and spleen tissues were then isolated. To avoid RNA degradation in the wild situation as much as possible and harvest high-quality nuclei for downstream library construction, all the tissues were snap-frozen in liquid nitrogen within 15 minutes. After analysis with a nucleic acid detection kit for coronavirus, the samples that were negative for coronaviruses were transferred to BGI-Shenzhen for subsequent experiments. This study was reviewed and approved by the Institutional Review Board of the Ethics Committee of BGI.

### Single-nucleus suspension preparation

The nuclei were extracted from each snap-frozen tissue following the protocol described previously^17^ with minor modifications. In brief, frozen tissues were thawed in a 30 mm culture dish and cut to a size of 1–3 mm^3^ with tweezers and scissors. The cut tissues were then transferred into a douncer (Kimble Chase, #885301–0002) with 2 mL chilled homogenization buffer consisting of 10 mM Trizma Hydrochloride Solution, pH 8.0, (Sigma-Aldrich, #T2694), 250 mM sucrose (Sangon Biotech, #A610498-0500), 25 mM KCl (Sigma-Aldrich, #60142-100ML-F), 5 mM MgCl_2_ (Ambion, #AM9530G), 0.1 mM DTT (Thermo Fisher Scientific, #18064014), 1X cOmplete™ Protease Inhibitor Cocktail (Roche, #04693116001), 0.4 U/μL RNase inhibitor (New England Biolabs, #M0314L), 0.1% Nonidet P40 Substitute (Roche, #11332473001) and 1% BSA (Sangon Biotech, #A600332-0005). The tissues were homogenized with 10 strokes of the loose pestle. After straining through a 70 μm cell strainer (Falcon, #352350), the homogenate was transferred to another douncer. Five strokes were applied with a loose pestle to completely release the nuclei. The homogenate was filtered through a 30 μm cell strainer (Sysmex, #04-004-2326). The nuclei were centrifuged at 500 × *g* for 5 minutes at 4 °C and then washed with 1X PBS supplemented with 1% BSA and 0.2 U/μL RNase inhibitor. Finally, the nuclei were collected by centrifugation and resuspended in cell resuspension buffer. The nuclei from each tissue were stained with DAPI (Beyotime, #C1006) and counted under a fluorescence microscope.

### snRNA-seq library construction

snRNA-seq libraries were constructed according to instructions of the DNBelab C4 scRNA Preparation Kit as previously described^17^. In short, the nuclei were diluted to a concentration of 1,000 nuclei/μL and loaded into the cell reservoir of a microfluidics chip. Barcoded beads and droplet generation oil were successively added to the beads and oil reservoirs. Encapsulated droplets were generated and collected in the DNBelab C4 system. The beads capturing mRNA were recovered for reverse transcription. After amplification by polymerase chain reaction, the cDNA was purified and quantified utilizing a Qubit^TM^ dsDNA kit (Invitrogen, #Q32854). Libraries of 3-end transcripts were subsequently constructed according to the manufacturer’s protocol, including cDNA fragmentation, size selection, end repair and A-tailing, adapter ligation, PCR for indexing libraries, and cyclization of the sequencing libraries. The sequencing libraries were purified and quantified with a Qubit^TM^ ssDNA kit (Thermo Fisher Scientific, #Q10212).

### snATAC-seq library construction

snATAC-seq libraries were prepared with the DNBelab C4 scATAC Library Preparation Kit as previously described^18^. In brief, the extracted nuclei were treated with a Tn5 transposase coupling adapter. The transposed nuclei and barcoded beads were encompassed in droplets by the DNBelab C4 system. Preamplification, the collection of beads capturing ATAC fragments, and secondary amplification were then successively carried out for the indexed sequencing libraries according to the manufacturer’s protocol. The sequencing libraries were quantified with a Qubit ssDNA Assay Kit.

### Sequencing

Both the snRNA-seq and snATAC-seq libraries were sequenced on the BGI DNBSEQ^TM^ technology platform. DNA nanoballs (DNBs) were generated from the libraries and loaded into patterned nanoarrays. The libraries were then sequenced on a sequencer according to the paired-end strategy. The read length of snRNA-seq libraries was 30 bp for read 1 and 100 bp for read 2. The snATAC-seq libraries contained 50 bp paired-end reads, and the barcode reads were 20 bp.

### snRNA-seq data processing

Raw reads were aligned to the genome of *Rhinolophus sinicus*^19^, and unique molecular identifiers (UMIs) count matrix was generated by using Cell Ranger (version 6.1.2). Reads were aligned to reference genome by Cell Ranger build-in STAR alignment pipeline, and all parameters are default values. We applied DoubletFinder^20^ to remove doublets for approximately 5% of the estimated total nuclei. Table 1 summarizes sequencing parameters for the snRNA-seq dataset. Consistent with the previous study^21^, the libraries from the same tissue were then analyzed using reciprocal PCA (RPCA) of Seurat (version 4.0.5) (https://satijalab.org/seurat/articles/integration_rpca.html) and Harmony (version 1.0) (https://portals.broadinstitute.org/harmony/) with default parameters to remove potential batch effects. The datasets of this study originate from the same platform better suitable for RPCA-based integrative analysis. Also, the marker genes identified by RPCA were slightly more specific than Harmony (Supplementary Fig. 1, Supplementary Table 1). Ultimately we chose the batch-correction result of the RPCA method for downstream analysis. Batch effect removal is essential in data integration analysis. Therefore, we recommend selecting a suitable method for data generating from different platforms after systematically evaluating different batch-correction methods.

**Table 1:** Overview of the QC summary for snRNA-seq libraries established for seven organs.

Organ	Library	Number of nuclei	Number of reads	Mean reads per nucleus	Total genes detected
Brain	Brain_RNA_1	3,151	675,207,398	179,121	16,790
Brain	Brain_RNA_2	3,635	400,798,751	104,511	16,815
Brain	Brain_RNA_3	2,572	306,883,628	199,706	16,681
Brain	Brain_RNA_4	2,123	686,226,480	267,059	16,559
Brain	Brain_RNA_5	2,285	732,754,162	265,960	16,552
Brain	Brain_RNA_6	2,328	604,901,073	211,935	16,703
Heart	Heart_RNA_1	2,906	648,267,846	148454	15,553
Heart	Heart_RNA_2	2,899	394,625,818	140,046	15,394
Heart	Heart_RNA_3	3,156	903,945,581	188,113	15,592
Heart	Heart_RNA_4	3,619	706,606,267	143,969	15,882
Heart	Heart_RNA_5	3,070	376,958,680	94,139	15,628
Kidney	Kidney_RNA_1	2,440	570,467,014	151,066	15,491
Kidney	Kidney_RNA_2	1,938	475,658,445	161,043	15,225
Kidney	Kidney_RNA_3	1,505	388,127,998	122,021	14,818
Kidney	Kidney_RNA_4	1,483	407,346,630	160,860	14,886
Kidney	Kidney_RNA_5	1,828	274,259,992	57,282	15,075
Liver	Liver_RNA_1	3,603	603,205,455	148,041	14,956
Liver	Liver_RNA_2	3,439	620,708,962	155,876	15,142
Liver	Liver_RNA_3	4,258	805,416,653	167,233	15,088
Liver	Liver_RNA_4	3,696	771,255,727	178,503	15,379
Liver	Liver_RNA_5	2,818	742,462,768	226,808	15,033
Liver	Liver_RNA_6	4,235	868,073,242	177,023	15,347
Lung	Lung_RNA_1	1,511	522,328,340	259,814	15,041
Lung	Lung_RNA_2	1,113	637,869,760	396,123	15,268
Lung	Lung_RNA_3	1,330	580,980,205	282,364	14,854
Lung	Lung_RNA_4	1,199	580,980,205	279,372	14,735
Spleen	Spleen_RNA_1	2,042	303,407,712	239,341	15,257
Spleen	Spleen_RNA_2	1,722	338,028,781	144,115	15,145
Spleen	Spleen_RNA_3	1,815	696,824,675	237,428	15,008
Spleen	Spleen_RNA_4	2,196	759,332,821	227,310	15,249
Spleen	Spleen_RNA_5	1,825	723,202,467	296,356	15,318
Spleen	Spleen_RNA_6	1,845	796,406,863	247,936	15,140
Stomach	Stomach_RNA_1	1,229	393,944,248	79,000	12,790
Stomach	Stomach_RNA_2	823	367,730,653	97,234	11,967
Stomach	Stomach_RNA_3	947	317,840,897	86,616	12,309
Stomach	Stomach_RNA_4	1,087	650,210,481	165,925	12,577
Stomach	Stomach_RNA_5	1,353	790,157,113	188,418	13,472
Stomach	Stomach_RNA_6	799	365,346,210	124,219	12,467

### Cell clustering and identification of cell types

Clustering analysis of the seven *Rhinolophus affinis* tissue datasets was performed using Seurat (version 4.0.5)^22^ in the R environment. The parameters of each function were manually curated to portray the optimal clustering of cells. In preprocessing, cells were filtered based on the distribution of genes and UMIs for each tissue. The criteria were as follows: (i) for the brain, heart, kidney, lung, liver, and spleen, a cell expressing a minimum of 200 genes and a gene that was expressed in a minimum of 3 nuclei; (ii) for the stomach, a cell expressing a minimum of 100 genes and a gene that was expressed in a minimum of 3 nuclei; (iii) for all tissues, a cell expressing a maximum of 2,500 genes. The filtered data were normalized and scaled according to Seurat NormalizeData and ScaleData with the default parameters. A total of 2,000 highly variable genes were selected for subsequent analysis. Dimension reduction starts with principal component analysis (PCA), and the number of principal components used for Uniform Manifold Approximation and Projection (UMAP) depends on the importance of the embeddings. The chosen resolution of the Louvain method was 0.4 for each tissue and 1.2 for all tissue together according to subgroup rationality. The results of the Louvain method distinguishing differential genes among clusters were ranked (Benjamini-Hochberg, Wilcoxon rank-sum test). Finally, we annotated each cell type according to extensive literature review and searching for specific gene expression patterns^9,10,23^.

### Expression analysis of virus receptor genes

45 bat virus receptor genes were collected from the public database. The results of the expression analysis of virus receptor genes were visualized by using Seurat and R.

### snATAC-seq data processing

Raw reads were split into insertions and barcodes and filtered by PISA (version 1.1) (https://github.com/PolyTTT/PISA/)^24^ with a minimum sequencing quality of 20. Table 2 summarized the sequencing parameters of the snATAC-seq datasets. Filtered reads were aligned to the *Rhinolophus sinicus* genome by BWA (version 0.7.17-r1188)^25^. BAM files were processed with bap2 (version 0.6.2)^26^, which can find barcodes from the same cell.

**Table 2:** Overview of the QC summary for snATAC-seq libraries established for kidney and lung.

Organ	Library	Number of nuclei	Number of reads	Mean fragments per nucleus
Lung	Lung _ATAC_1	3,398	565,363,782	14,220
Lung	Lung _ATAC_2	2,231	475,006,820	18,740
Kidney	Kidney _ATAC_1	3,342	610,657,860	9,643
Kidney	Kidney _ATAC_2	3,707	634,592,167	8,672

### snATAC-seq data analysis

Files of accessible read fragments were generated by using bap2 software. Downstream ATAC-seq data analysis was performed with ArchR^27^. The offset of the positive chain Tn5 insertion was +4, while that of the negative chain was −5. The promoter region was 2,000 bp upstream and 100 bp downstream of the transcription start sites (TSSs). We used the following selection criteria to filter out low-quality cells: (i) We filtered out all single nuclei that had fewer than 3,004 and 4,471 unique fragments in the kidney and lung, respectively; (ii) Single nuclei less than 5.149 and 6.409 TSS enrichment scores were filtered out in the kidney and lung, respectively; (iii) Potential diploids were further removed based on the ArchR method (filterRatio = 2.5). The batch effect of libraries originating from same tissue was corrected by using Harmony^28^. Dimensionality reduction was performed using iterative latent semantic indexing (LSI) in ArchR, and clustering was performed using the Leiden algorithm based on Seurat (resolution = 0.5). snATAC-seq cell-type labels were identified according to classical markers. We used the addGeneIntegrationMatrix function to integrate snATAC-seq dataset with snRNA-seq dataset with unconstrained methods in ArchR. After labeling the cell types in each dataset, the peaks of each cell type were generated using addGroupCoverages function and addReproduciblePeakSet function. We identified differentially accessible peaks (DAPs) in an unsupervised fashion in ArchR using the addMarkerFeatures function. DAPs were selected with the filter string “FDR ≤ 0.1 & Log2FC ≥ 0.5”.

## Data Records

All raw data have been submitted to the CNGB Nucleotide Sequence Archive (https://db.cngb.org/search/project/CNP0001406/)^29^. Raw data have also been submitted to the NCBI Sequence Read Archive, and the BioProject accession identifier is PRJNA693364^30^. The cell-gene matrix, cell-peak matrix and all cell cluster files were submitted to the CNGB Nucleotide Sequence Archive (https://db.cngb.org/search/project/CNP0001406/)^29^.

## Technical Validation

### snRNA-seq

**Fig. 1:**
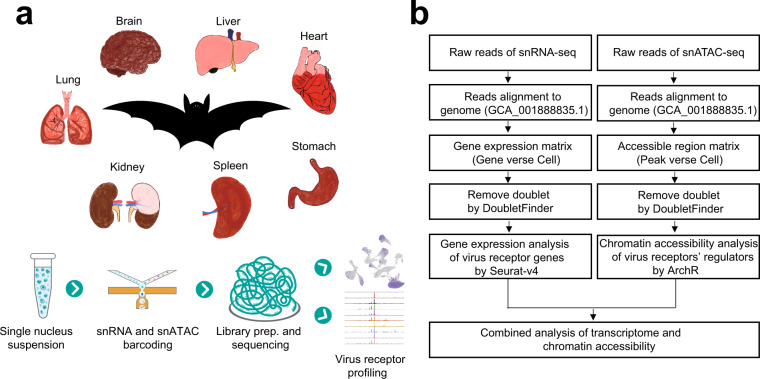
Experimental design and analysis pipeline. (**a**) A schematic representation of the bat organs evaluated in this study and experimental design for single-nucleus sequencing. (**b**) Data processing pipeline for snRNA-seq data and snATAC-seq data.

**Fig. 2:**
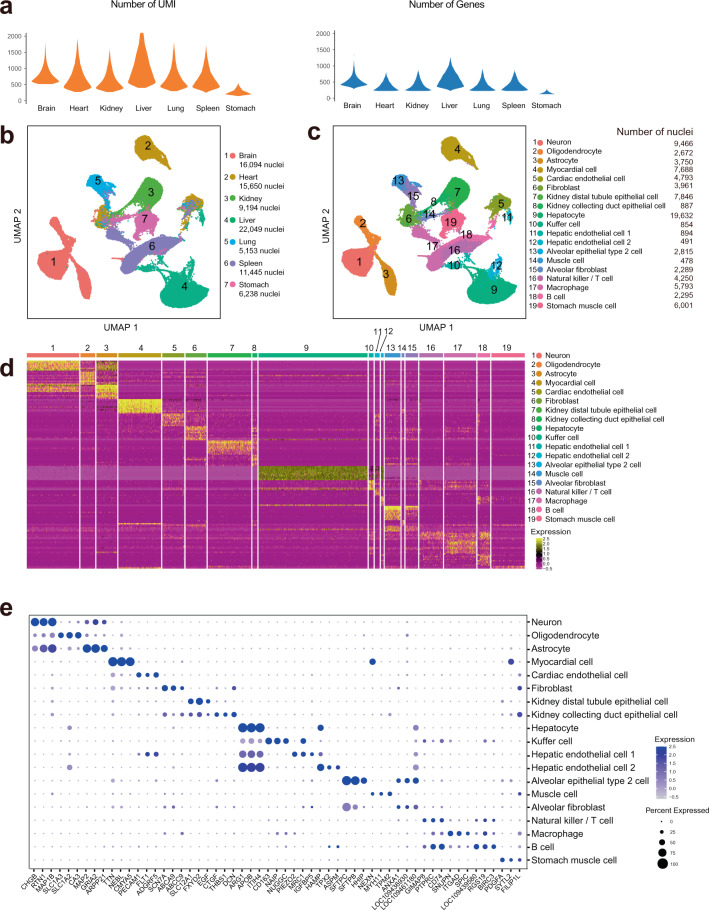
snRNA-seq data quality control and features. (**a**) Violin plot showing the numbers distribution of UMIs (left) and genes (right) in each organ. (**b**) UMAP showing all single-cell patterns in 2D space, colored according to the organ. The numbers of nuclei in each organ are listed. (**c**) UMAP showing all single-cell patterns in 2D space, colored according to Louvain clusters. Cell type annotation and cell numbers for each cluster are listed. (**d**) Heatmap showing the marker genes expression pattern of each cluster using the scaled expression value. Corresponding cluster annotations are listed. (**e**) Dotplot plot showing representative markers expression patterns, which were used for annotating clusters.

Nuclei were extracted from the organ specimens isolated from bats, and single-nucleus suspensions were then prepared for snRNA-seq and snATAC-seq (see methods) (Fig. 1a). The data were processed via a standard pipeline (Fig. 1b). Samples from seven major organs were prepared for snRNA-seq, including the brain, heart, kidney, liver, lung, spleen and stomach. After quality control (see methods), we obtained a total of 85,832 nuclei; the mean UMIs for each nucleus was 901 (Fig. 2a), and the mean gene number for each nucleus was 488 (Fig. 2a). The stomach exhibited far fewer UMIs and genes than other organs, but considering that the stomach tissue data showed significantly related markers, we decided to retain the data (Supplementary Fig. 2). UMAP showed that the cells from different organs were clearly separated, although a small number of mixed cells were observed (Fig. 2b). Using the unsupervised cluster algorithm Louvain (see methods), we obtained 19 clusters (Fig. 2c), and each cluster included significant specific gene sets (Fig. 2d, Supplementary Table 1). We annotated each cluster to corresponding cell types according to its specific markers (Fig. 2e). The results for each organ could be further clustered into several specific subgroups (Supplementary Fig. 2) with significant distinct markers (Supplementary Fig. 3).

### snATAC-seq

**Fig. 3:**
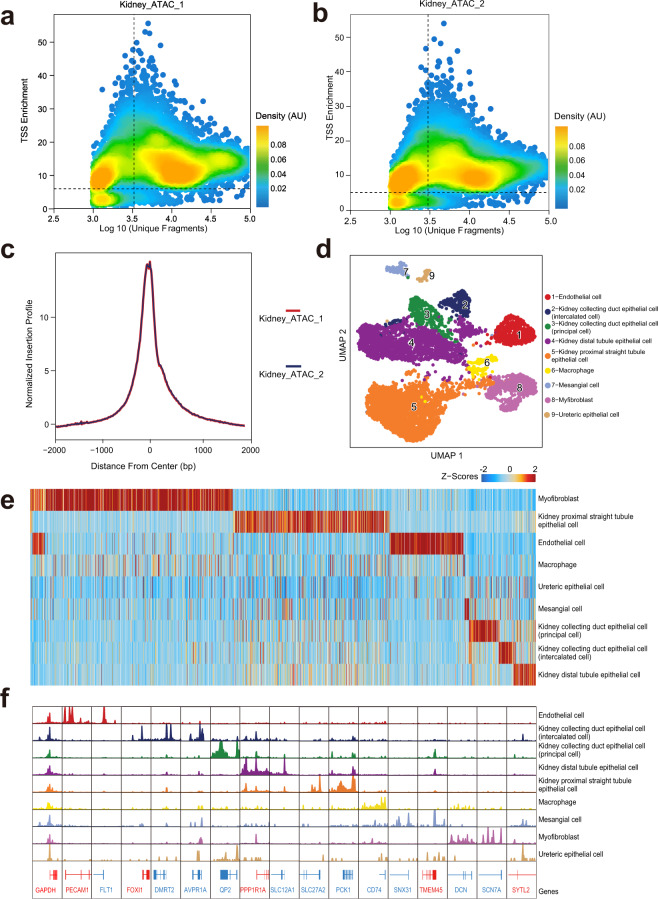
Bat kidney snATAC-seq data quality control and features. (**a,b**) QC filtering plots from ArchR showing the TSS enrichment scores of Kidney_ATAC_1 and Kidney_ATAC_2. (**c**) Plot showing the normalized insertion profile around the TSSs of two kidney libraries. (**d**) UMAP showing the cell distribution pattern in 2D space, colored according to Louvain clusters. (**e**) Heatmap representing chromatin accessibility in binarized peaks from the kidney peak set. Each row represents an individual pseudobulk of each cell type, and each column represents a peak, colored according to the column z-scores. (**f**) Aggregated chromatin accessibility profiles of each cell type at representative marker gene loci.

SARS-CoV-2 is mainly detected in the lung and kidney^31,32^, so we sought to explore the chromatin accessibility of related virus receptors in these two organs. Hence, we performed snATAC-seq of the kidney and lung and obtained two libraries for each organ. After quality control, we obtained 7,049 nuclei from the kidney and 5,629 nuclei from the lung. In the kidney dataset, the two libraries showed similar quality features, the TSS enrichment scores were mostly distributed between 8–25, and the numbers of unique nuclear fragments were mainly distributed from 6,000–60,000 (Fig. 3a,b). The TSS enrichment profile showed a clear peak at the TSS and a smaller peak caused by a well-positioned + 1 nucleosome to the right of the center (Fig. 3c). The results showed that the two libraries presented similar performance. Then, nuclei were clustered and annotated by using ArchR (see methods), which covered 9 kidney cell types (Fig. 3d). Using these clusters, we called peaks to create a union set of 266,110 reproducible peaks based on pseudobulk chromatin accessibility. Peaks of specific expression were identified in all clusters (Fig. 3e,f). We performed the same analysis on the lung dataset, and we obtained 5,629 nuclei, which were clustered and annotated to six lung cell types, a total of 267,879 peaks were called (Supplementary Fig. 4).

### Consistency of snRNA-seq and snATAC-seq results

**Fig. 4:**
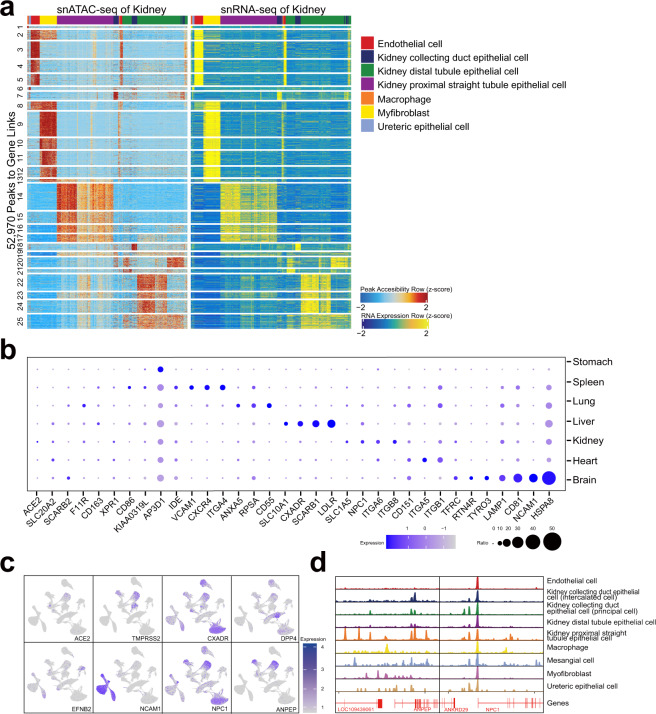
Bat virus receptor expression patterns and chromatin accessibility profiles across organs. (**a**) Heatmap of peak-to-gene links in the kidney generated using ArchR. (**b**) Selected bat virus receptor expression patterns among organs, including organ-specific expression and general expression. For the receptors with organ-specific expression, the expression patterns in the corresponding organs among different cell types are shown in Supplementary Fig. 6. (**c**) Representative well-studied bat virus receptor expression pattern drawn on the UMAP related to Fig. 2b. (**d**) Chromatin accessibility of two representative genes in different kidney cell types.

Next, we integrated the snRNA-seq and snATAC-seq data. To visualize the correspondence between peaks and genes, we generated a peak-to-gene heatmap containing two side-by-side heatmaps, one of which represented the snATAC-seq data, while the other represented the snRNA-seq data. We identified 52,970 and 84,651 peak-to-gene links in the kidney and lung, respectively, in which the results showed strong consistency between the peaks and genes (Fig. 4a, Supplementary Fig. 5a).

### BAT-related virus receptor features

We checked the relationship between the expression profile and chromatin accessibility of representative virus receptor genes. We found that *CD55* was mostly expressed in the lung and was not expressed in the kidney (Supplementary Fig. 5b), and *CD55* showed specific peaks near the TSS in the lung (Supplementary Fig. 5c). We systematically checked the expression patterns of bat virus receptors in our data. First, we collected data on 102 bat virus receptors using the DBatVir database^16^ and public datasets, among 25 receptors were expressed in organ-specific patterns (Fig. 4b, Supplementary Table 2). Furthermore, most of these 25 receptors were expressed only in specific cell types in the corresponding organs (Supplementary Fig. 6).

Bats have been identified as natural reservoir hosts for several emerging viruses that can cause severe disease in humans, including Ebola, SARS-CoV, MERS-CoV, etc. Therefore, we checked the expression pattern of 8 bat receptors of zoonotic viruses^23^, including *ACE2*, *TMPRSS2* (related to SARS-CoV, SARS-CoV-2)^33,34^, *DPP4* (related to MERS-CoV)^35^, *ANPEP* (related to HCoV-229E)^36^, *EFNB2* (related to Hendra virus and Nipah virus)^37^, *NPC1* (related to Ebola virus and Marburg virus)^38^, *CXADR* (related to adenovirus)^39^, and *NCAM1* (related to rabies virus)^40^. The results showed that *ACE2* was weakly expressed in the kidney and spleen, whereas other receptors were expressed in an organ- and cell-type-specific manner (Fig. 4c). Then, we checked the chromatin accessibility of several representative receptors and found that *ANPEP* and *NPC1* were selectively expressed in several cell types in the kidney (Supplementary Fig. 6) and that the peaks located near their TSSs also showed a cell type-specific pattern (Fig. 4d).

We provided high-quality snRNA-seq data and snATAC-seq data from several major organs of *Rhinolophus affinis*. We systematically screened the expression pattern and accessibility features of bat virus receptors, which will provide a valuable resource for further research on the pathogenesis and zoonotic transmission of bat-borne viruses.

## Usage Notes

The snRNA-seq data analyses, including the processing pipeline, read mapping, gene calling, and the snATAC-seq data processing pipeline, including read mapping and peak calling, were run on the Linux operating system. All R source codes with the optimized parameters used for the downstream data analyses and visualization are provided online (https://figshare.com/s/132dd4a1d364e459bac8)^41^.

## Supplementary information

Supplementary Table 1

SUPPLEMENTARY INFORMATION

Supplementary Table 2

## Data Availability

The R code used to identify cell subclusters and profile tissue-specific virus receptor expression and tissue-specific chromatin accessible regions are available online (https://figshare.com/s/132dd4a1d364e459bac8)^41^.
