# Supplementary material for: A map of bat virus receptors derived from single-cell multiomics
Source: Sci Data. 2022 Jun 14;9:336. doi: 10.1038/s41597-022-01447-7 (PMC9195401; doi:10.1038/s41597-022-01447-7)
Supplement: Supplementary file 2 — SUPPLEMENTARY INFORMATION [file 41597_2022_1447_MOESM2_ESM.pdf]

Supplementary information

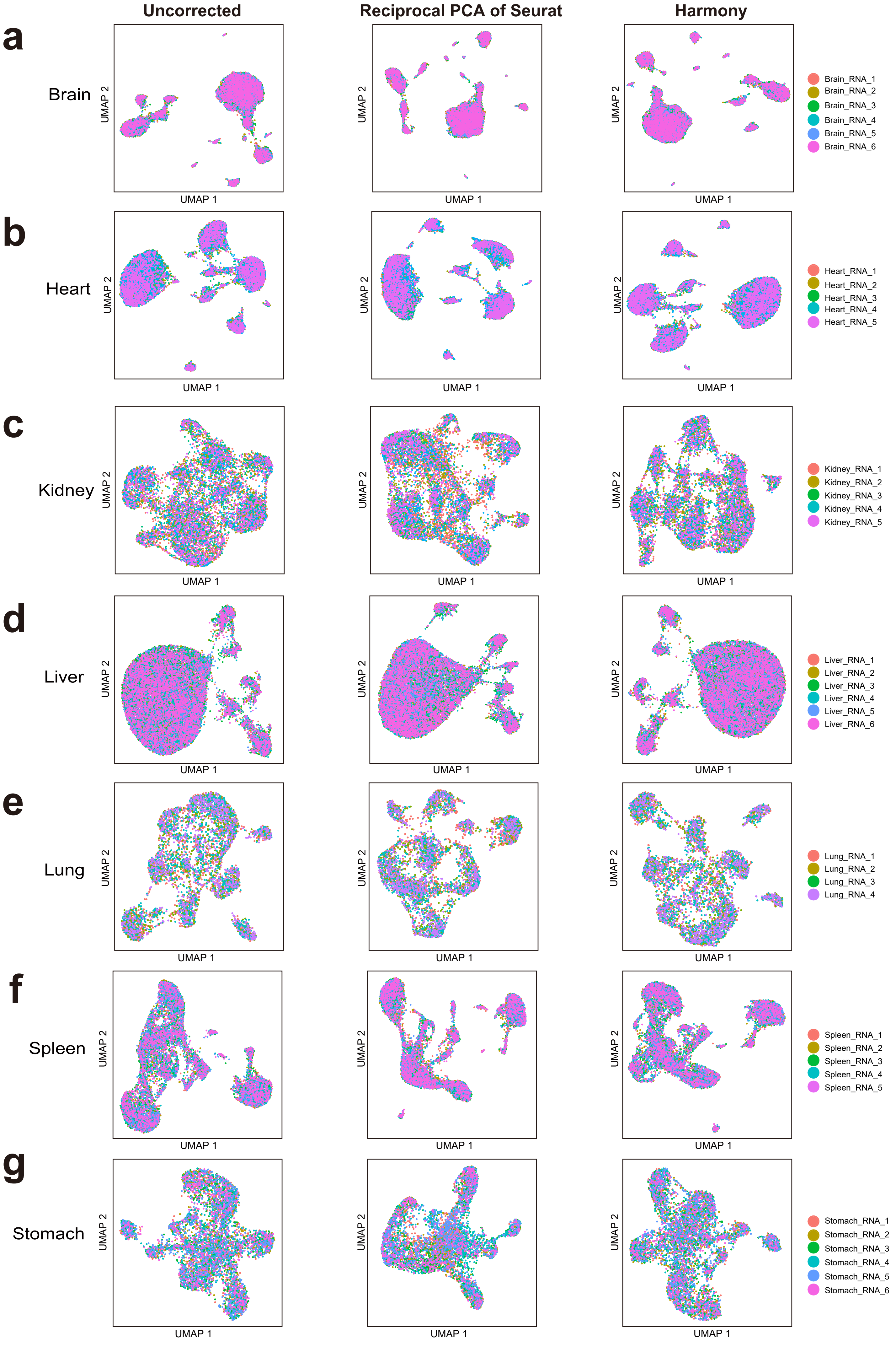

Supplementary Figure 1. UMAP visualization for each organ processing by different batch effect correction methods, uncorrected (left panel), Reciprocal PCA of Seurat (middle panel), and Harmony (right panel), colored according to libraries. The librari-es' IDs are listed on the right (**a**-**g**).

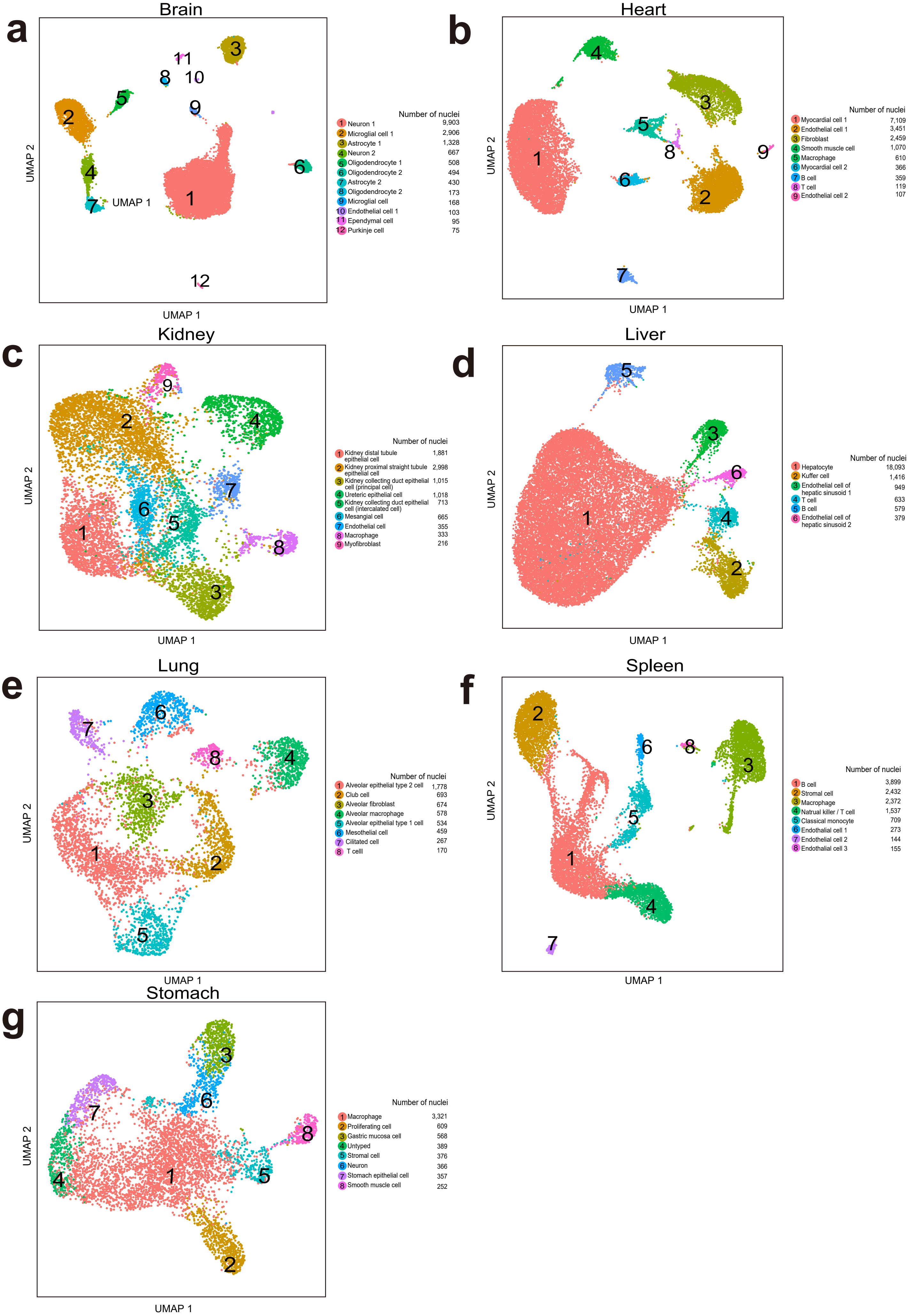
a
Brain
UMAP 1
UMAP 2
Number of nuclei
1 Neuron 1 9,903
2 Microglial cell 1 2,906
3 Astrocyte 1 1,328
4 Neuron 2 667
5 Oligodendrocyte 1 508
6 Oligodendrocyte 2 494
7 Astrocyte 2 430
8 Oligodendrocyte 2 173
9 Microglial cell 168
10 Endothelial cell 1 103
11 Ependymal cell 95
12 Purkinje cell 75
b
Heart
UMAP 1
UMAP 2
Number of nuclei
1 Myocardial cell 1 7,109
2 Endothelial cell 1 3,451
3 Fibroblast 2,459
4 Smooth muscle cell 1,070
5 Macrophage 610
6 Myocardial cell 2 366
7 B cell 359
8 T cell 119
9 Endothelial cell 2 107
c
Kidney
UMAP 1
UMAP 2
Number of nuclei
1 Kidney distal tubule epithelial cell 1,881
2 Kidney proximal straight tubule epithelial cell 2,998
3 Kidney collecting duct epithelial cell (principal cell) 1,015
4 Ureteric epithelial cell 1,018
5 Kidney collecting duct epithelial cell (intercalated cell) 713
6 Mesangial cell 665
7 Endothelial cell 355
8 Macrophage 333
9 Myofibroblast 216
d
Liver
UMAP 1
UMAP 2
Number of nuclei
1 Hepatocyte 18,093
2 Kuffer cell 1,416
3 Endothelial cell of hepatic sinusoid 1 949
4 T cell 633
5 B cell 579
6 Endothelial cell of hepatic sinusoid 2 379
e
Lung
UMAP 1
UMAP 2
Number of nuclei
1 Alveolar epithelial type 2 cell 1,778
2 Club cell 693
3 Alveolar fibroblast 674
4 Alveolar macrophage 578
5 Alveolar epithelial type 1 cell 534
6 Mesothelial cell 459
7 Cilitated cell 267
8 T celll 170
f
Spleen
UMAP 1
UMAP 2
Number of nuclei
1 B cell 3,899
2 Stromal cell 2,432
3 Macrophage 2,372
4 Natrual killer / T cell 1,537
5 Classical monocyte 709
6 Endothalial cell 1 273
7 Endothelial cell 2 144
8 Endothalial cell 3 155
g
Stomach
UMAP 1
UMAP 2
Number of nuclei
1 Macrophage 3,321
2 Proliferating cell 609
3 Gastric mucosa cell 568
4 Untyped 389
5 Stromal cell 376
6 Neuron 366
7 Stomach epithelial cell 357
8 Smooth muscle cell 252

Supplementary Figure 2. UMAP visualization for each organ, colored according to and Louvain clusters. The cell type annotation and cell numbers are listed on the right (**a**-**g**).

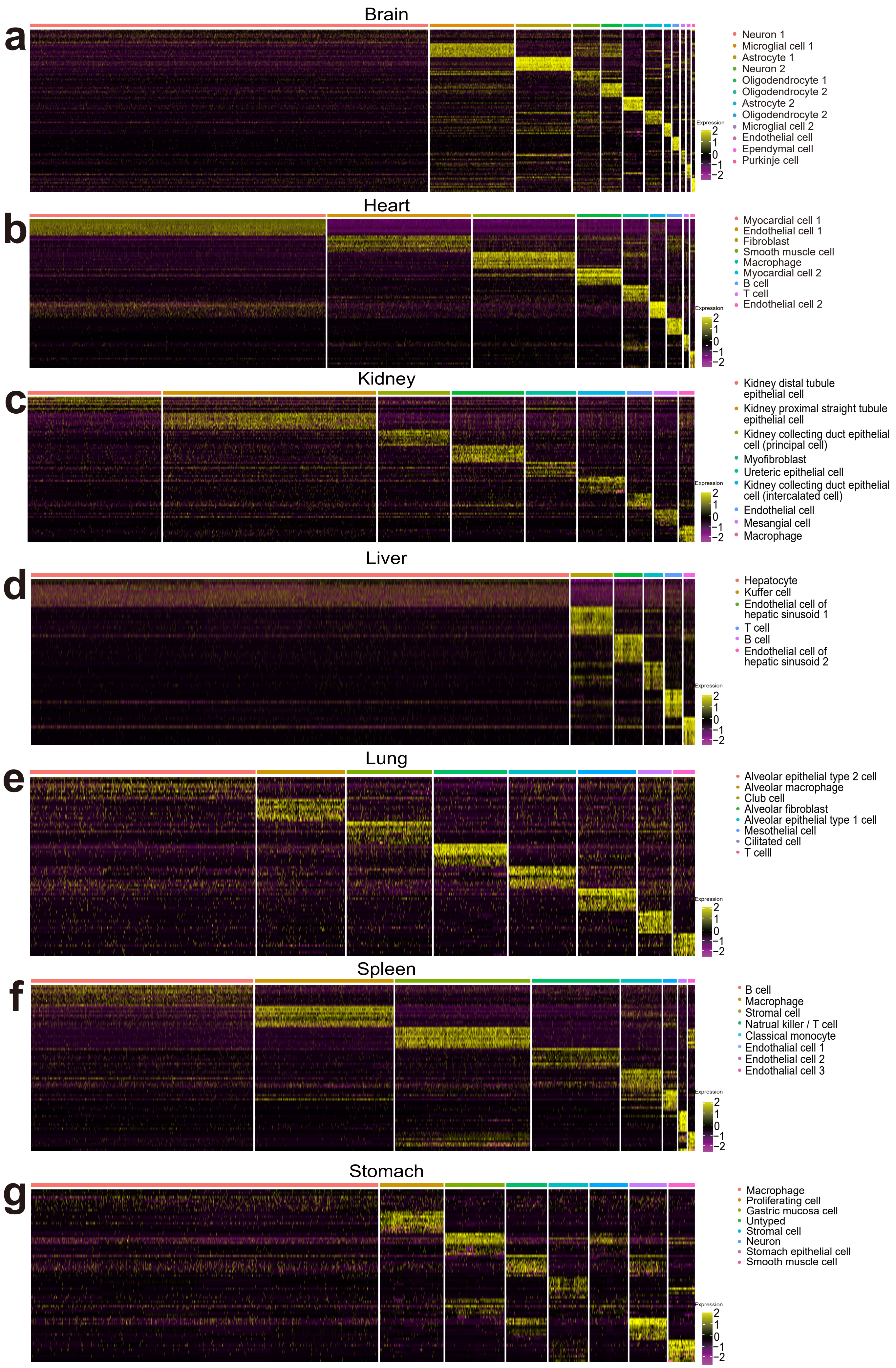
a
Brain
Expression
2
1
0
−1
−2
Neuron 1
Microglial cell 1
Astrocyte 1
Neuron 2
Oligodendrocyte 1
Oligodendrocyte 2
Astrocyte 2
Oligodendrocyte 2
Microglial cell 2
Endothelial cell
Ependymal cell
Purkinje cell
b
Heart
Myocardial cell 1
Endothelial cell 1
Fibroblast
Smooth muscle cell
Macrophage
Myocardial cell 2
B cell
T cell
Endothelial cell 2
c
Kidney
Kidney distal tubule epithelial cell
Kidney proximal straight tubule epithelial cell
Kidney collecting duct epithelial cell (principal cell)
Myofibroblast
Ureteric epithelial cell
Kidney collecting duct epithelial cell (intercalated cell)
Endothelial cell
Mesangial cell
Macrophage
d
Liver
Hepatocyte
Kuffer cell
Endothelial cell of hepatic sinusoid 1
T cell
B cell
Endothelial cell of hepatic sinusoid 2
e
Lung
Alveolar epithelial type 2 cell
Alveolar macrophage
Club cell
Alveolar fibroblast
Alveolar epithelial type 1 cell
Mesothelial cell
Cilitated cell
T celll
f
Spleen
B cell
Macrophage
Stromal cell
Natrual killer / T cell
Classical monocyte
Endothalial cell 1
Endothalial cell 2
Endothalial cell 3
g
Stomach
Macrophage
Proliferating cell
Gastric mucosa cell
Untyped
Stromal cell
Neuron
Stomach epithelial cell
Smooth muscle cell

Supplementary Figure 3. Heatmap of marker genes for each subtype in specific organs, corresponding to Supplementary Figure 2.

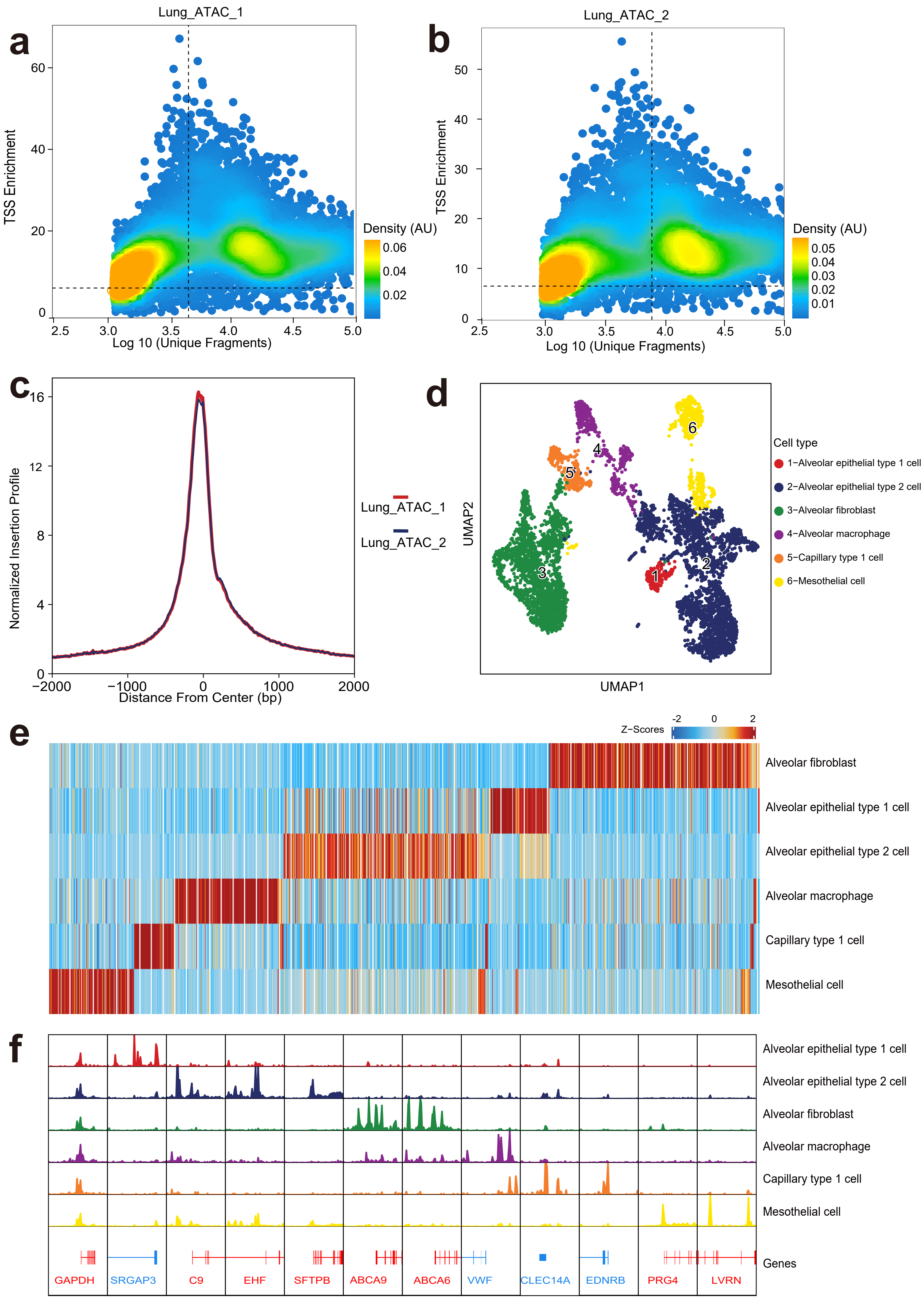

a
Lung_ATAC_1
TSS Enrichment
Log 10 (Unique Fragments)
Density (AU)
b
Lung_ATAC_2
TSS Enrichment
Log 10 (Unique Fragments)
Density (AU)
c
Normalized Insertion Profile
Distance From Center (bp)
Lung_ATAC_1
Lung_ATAC_2
d
UMAP2
UMAP1
Cell type
1−Alveolar epithelial type 1 cell
2−Alveolar epithelial type 2 cell
3−Alveolar fibroblast
4−Alveolar macrophage
5−Capillary type 1 cell
6−Mesothelial cell
e
Z−Scores
Alveolar fibroblast
Alveolar epithelial type 1 cell
Alveolar epithelial type 2 cell
Alveolar macrophage
Capillary type 1 cell
Mesothelial cell
f
Alveolar epithelial type 1 cell
Alveolar epithelial type 2 cell
Alveolar fibroblast
Alveolar macrophage
Capillary type 1 cell
Mesothelial cell
Genes
GAPDH
SRGAP3
C9
EHF
SFTPB
ABCA9
ABCA6
VWF
CLEC14A
EDNRB
PRG4
LVRN

Supplementary Figure 4. Bat lung snATAC-seq data quality control and features. (**a**-**b**) QC filtering plots from ArchR showing the TSS enrichment scores of Lung_ATAC_1 and Lung_ATAC_2. (**c**) Plot showing the normalized insertion profile around the TSSs of two lung libraries. (**d**) UMAP showing the cell distribution pattern in 2D space, colored a-ccording to Louvain clusters. (**e**) Heatmap representing chromatin accessibility in binari-zed peaks from the lung peak set. Each row represents an individual pseudobulk of each cell type, and each column represents a peak, colored according to column z-scores. (**f**) Aggregated chromatin accessibility profiles of each cell type at representative marker ge-ne loci.

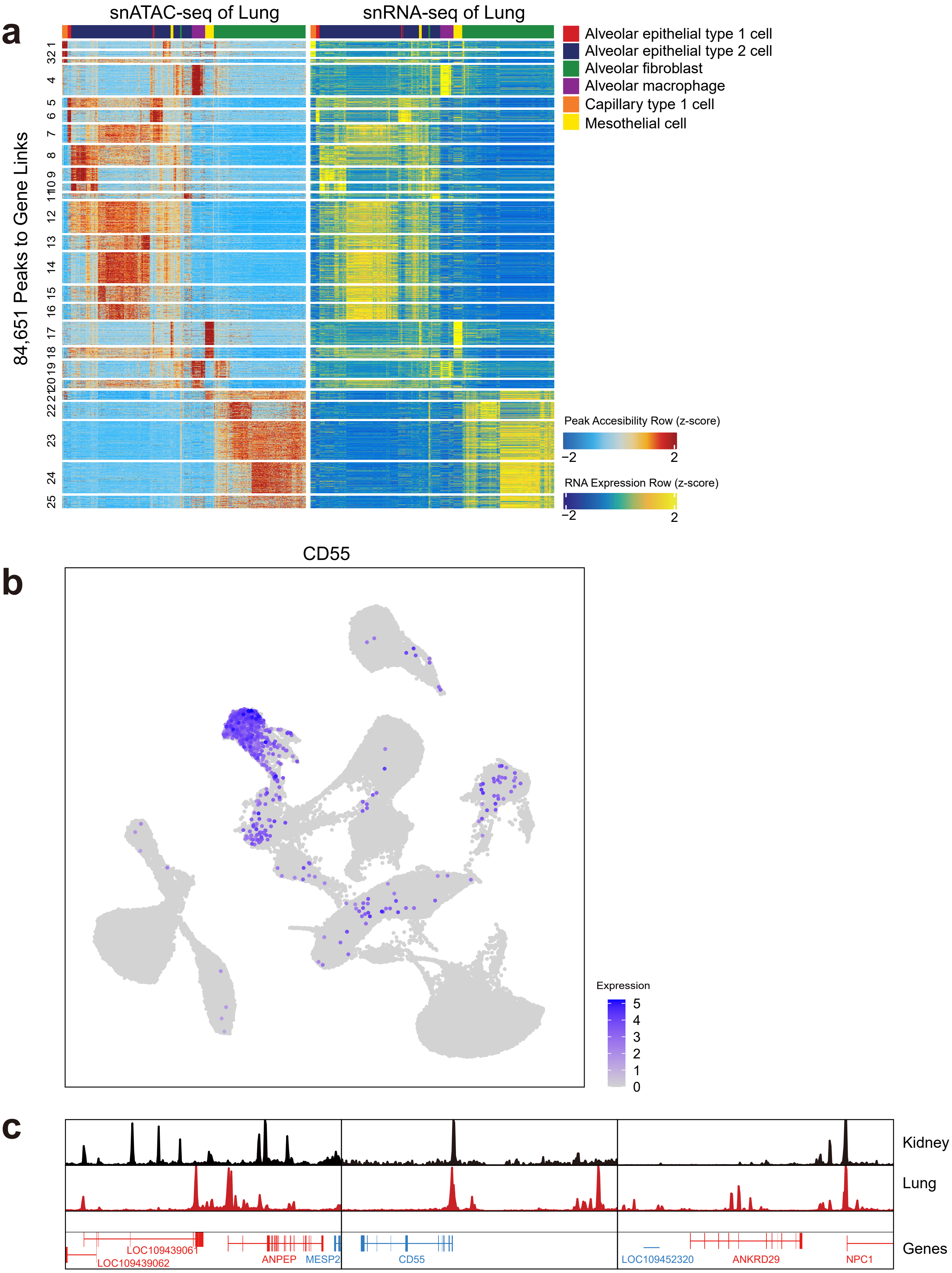
a
snATAC-seq of Lung
snRNA-seq of Lung
84,651 Peaks to Gene Links
Alveolar epithelial type 1 cell
Alveolar epithelial type 2 cell
Alveolar fibroblast
Alveolar macrophage
Capillary type 1 cell
Mesothelial cell
Peak Accesibility Row (z-score)
−2
2
RNA Expression Row (z-score)
−2
2
b
CD55
Expression
5
4
3
2
1
0
c
Kidney
Lung
Genes
LOC109439061
LOC109439062
ANPEP
MESP2
CD55
LOC109452320
ANKRD29
NPC1

Supplementary Figure 5. Combined analysis of snRNA-seq data and snATAC-seq data from the kidney and lung. (**a**) Heatmap of peak-to-gene links in the lungs generated using ArchR. (**b**) UMAP visualization of CD55 expression in all organs. (**c**) Chromatin accessibility of representative genes identified from pseudobulk ATAC-seq data of the kidney and lung.

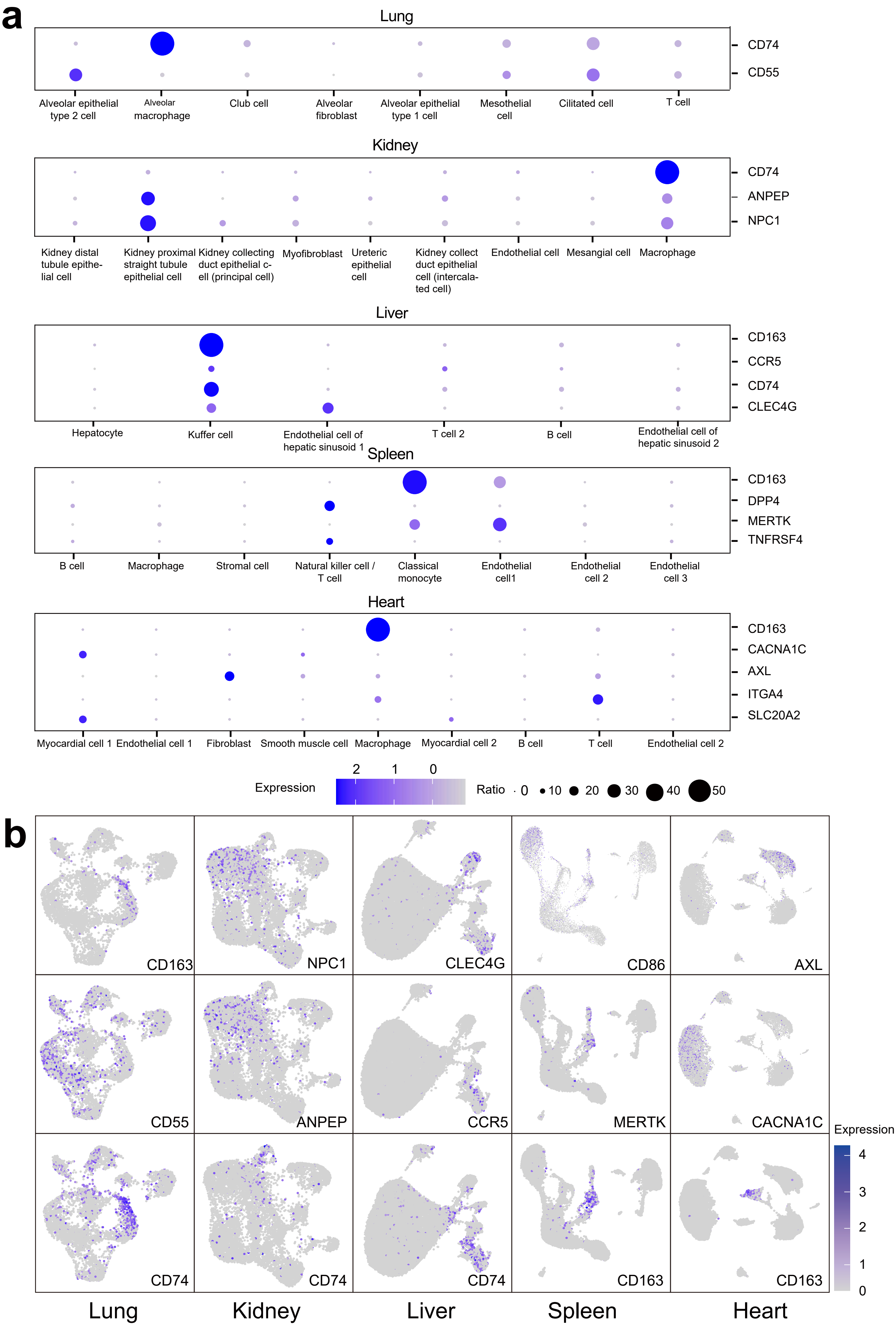
a
Lung
CD74
CD55
Alveolar epithelial type 2 cell
Alveolar macrophage
Club cell
Alveolar fibroblast
Alveolar epithelial type 1 cell
Mesothelial cell
Cilitated cell
T cell
Kidney
CD74
ANPEP
NPC1
Kidney distal tubule epithelial cell
Kidney proximal straight tubule epithelial cell
Kidney collecting duct epithelial cell (principal cell)
Myofibroblast
Ureteric epithelial cell
Kidney collect duct epithelial cell (intercalated cell)
Endothelial cell
Mesangial cell
Macrophage
Liver
CD163
CCR5
CD74
CLEC4G
Hepatocyte
Kuffer cell
Endothelial cell of hepatic sinusoid 1
T cell 2
B cell
Endothelial cell of hepatic sinusoid 2
Spleen
CD163
DPP4
MERTK
TNFRSF4
B cell
Macrophage
Stromal cell
Natural killer cell / T cell
Classical monocyte
Endothelial cell1
Endothelial cell 2
Endothelial cell 3
Heart
CD163
CACNA1C
AXL
ITGA4
SLC20A2
Myocardial cell 1
Endothelial cell 1
Fibroblast
Smooth muscle cell
Macrophage
Myocardial cell 2
B cell
T cell
Endothelial cell 2
Expression
2 1 0
Ratio 0 10 20 30 40 50
b
CD163
NPC1
CLEC4G
CD86
AXL
CD55
ANPEP
CCR5
MERTK
CACNA1C
CD74
CD74
CD74
CD163
CD163
Expression
4 3 2 1 0
Lung
Kidney
Liver
Spleen
Heart

Supplementary Figure 6. Organ-specific bat virus receptor genes expression pattern among cell types. Dotplot plot (**a**) and UMAP plot (**b**) showing representative gene expre-ssion patterns among cell types in each organ (Related to Figure 4c).
